# Competitive Fitness of *Mycobacterium tuberculosis in vitro*

**DOI:** 10.4103/ijmy.ijmy_97_19

**Published:** 2019

**Authors:** Ousmane Kodio, Antieme Combo Georges Togo, Yeya dit Sadio Sarro, Bintou Fane, Fatimata Diallo, Amadou Somboro, Boureima Degoga, Mahamadou Kone, Gagni Coulibaly, Mohamed Tolofoudje, Sidy Bane, Moumine Sanogo, Bourahima Kone, Nadie Coulibaly, Djeneba Dabitao, Bocar Baya, Mamoudou Maiga, Flabou Bougoudogo, Fasse Samake, Sounkalo Dao, Seydou Doumbia, Souleymane Diallo, Bassirou Diarra

**Affiliations:** 1 University Clinical Research Center, University of Sciences, Techniques and Technologies of Bamako, Bamako, Mali; 2 National Health Laboratory, Ministry of Health and Social Affairs, Bamako, Mali; 3 Center for Innovation in Global Health Technologies, Northwestern University, Feinberg School of Medicine, Chicago, IL, USA; 4 National Institute of Public Health Research, Ministry of Health and Social Affairs, Bamako, Mali; 5 Microbial Biotechnology Laboratory, University of Sciences, Techniques and Technologies of Bamako, Bamako, Mali

**Keywords:** Cost of resistance, *in vitro* competition, Mali, *Mycobacterium tuberculosis*-multidrug-resistant

## Abstract

**Background:**

While, bacteria resistance mutations can affect competitive fitness, given our multidrug-resistant (MDR) prevalence, we conducted this study to determine the impact of MDR on the competitive fitness of *Mycobacterium tuberculosis* (MTB) complex MDR strains. We conducted a cross-sectional study at the University Clinical Research Center (UCRC) from January to December 2017. New TB patients over aged of 18 were recruited at University teaching hospital and health reference centers of Bamako in USTTB Ethical committee approved protocols.

**Methods:**

MDR and drug-susceptible (wild-type [WT]) MTB strains (T1 and Beijing) and MTB H37Rv were competed on solid media in UCRC’s Tuberculosis Laboratory. Competitive and individual cultures were incubated for 14 days at 37°C with 7% CO2. Number of generation, generation time, and relative competitive fitness (W) of the strains were calculated. Data were analyzed with Epi-Info 7.1.5.2 software (CDC). *P* value was considered significant when it was <0.05. Scientific calculator (CS-82TL) was used for competitive fitness parameters calculations.

**Results:**

We performed 24 competitive cultures and 10 individual cultures. In individual cultures, strains’ generation number was for Beijing (WT: 4.60 and mutant MR: 4.40), T1 (WT: 2.69 and MR: 2.37), and H37Rv: 2.91. Generation number of WT strains was less than those of MDR strains in both individual and competitive culture. Relative competitive fitness was below 1 (W˂1) in 83.3%.

**Conclusion:**

MDR strains were less competitive than WT strains in 83.3% of cases. Resistant mutation impacts bacteria fitness.

## Introduction

Tuberculosis (TB) is an infectious disease in which causative agent is the *Mycobacterium tuberculosis* complex (MTBc). One-third of the world’s population is infected with MTB at an annual incidence rate of 10%. In 2016, 10.4 million new TB cases and 1.8 million deaths were reported.^[1]^ TB remains the most common of all potentially fatal bacterial infections worldwide and a major public health problem.^[1]^ Despite enormous efforts in the management of TB, the emergence of multidrug-resistant strains (MDR-TB: TB strain resistant to at least isoniazid and rifampicin) and ultra-resistant strains (strain of MDR-TB which is also resistant to fluoroquinolones and an injectable molecule) coupled with the HIV/AIDS pandemic complicates disease control. According to the WHO, 600,000 new cases of rifampicin resistance including 490,000 cases of MDR-TB and 1.2 million (11%) TB/HIV coinfection worldwide were reported in 2016.^[1]^

Antibiotic resistance in MTB results mainly in mutation of the targets gene of the antibiotic or by integration of plasmids. Antibiotics target essential cell functions and resistance mutations in these targeted genes may affect the biology or physiology of the bacteria, this is the concept of “cost of antibiotic resistance”. ^[2]^

Mathematical models predict that future of the epidemiology of MDR-TB and extensively DR-TB (XDR-TB) will depend on the competitive fitness of antibiotic-resistant strains relative to susceptible strains.^[3,4]^ Fitness of bacteria is its ability to replicate and survive in a competitive environment.^[5]^ According to the initial theory, mutations that lead to development of antibiotic resistance are considered a burden and are associated with reduced competitive fitness of the bacteria.^[6,7]^ This competitive fitness can be influenced not only by the mutation but also by genetic background of the strain.^[3]^ Some studies have shown that bacteria resistance to antibiotics generally confers a reduction in their competitive fitness, called “biological cost or cost of resistance” in the bacteria.^[2,3,7]^ However, in some cases, this may be at low or no cost in bacteria.^[3,8]^ The reduction in competitive fitness leads to an alteration of bacteria normal function, such as reduction of the rate of growth, virulence, and transmission.^[3,7]^ However, this cost can be offset by additional mutations called compensatory mutations.^[9–11]^

In Mali, the prevalence and incidence of TB were 91/100,000 inhabitants and 57/100,000 inhabitants, respectively, in 2016,^[1]^ with an increased rate of MDR-TB from 2.1% (2011) to 3.4% (2016) among new TB patients in Bamako region.^[12,13]^ Although the entire first six human MTBc lineages are represented in the population, plus *Mycobacterium bovis*, the “modern” lineage (L4) predominantly T1 genotype is the most prevalent and most associated with MDR (47.7%)^[12,14]^ and XDR (0.9%).^[12,15]^ The Beijing (L2) genotype reported in Mali in 2010^[16]^ is known to be associated with MDR and XDR and it is also indexed as hypervirulent worldwide.^[9,17]^ Its prevalence in Bamako region is about 1.8% and 33.3% of its strains were MDR.^[12]^

Given the increased prevalence of primary DR and the scarcity of fitness data on mycobacterial strains in TB-endemic countries, we conducted this study to determine the impact of MDR on the competitive fitness of MDR strains of both MTB T1 and Beijing genotypes relative to their drug-susceptible strains *in vitro* in Bamako.

## Methods

### Study design, period, and place

We conducted a prospective descriptive cross-sectional study conducted at the University Clinical Research Center (UCRC) BSL-3 Laboratory from January to December 2017. Samples previously stored in −80°C were thawed, subcultured on liquid and solid media, and tested for fitness assay. Those samples were collected from new and previously treated patients between 2010 and 2017 from Bamako region and enrolled in IRB-approved protocols.

### Culture, identification, first-line drug susceptibility testing, and strain typing

At the time of collection, samples were tested for culture, identification, first-line drug susceptibility testing and strain typing as previously described.^[12,16]^

### Competitive fitness

Two strains of MDR-TB genotype T1 and Beijing and two pan-sensitive to first-line antituberculous drugs (wild-type [WT]) were competed on Middlebrook 7H11 solid media to evaluate their growth. Twenty microliters of colonies of the strains was transferred into a tube containing six glass beads and 4 ml of physiological saline, then vortexed for 2–3 min, and allowed to sit for 20 min. The supernatant was transferred to another sterile dry tube without beads and allowed to sit for 15 min. This last supernatant was adjusted to the concentration of 1.5 × 10^8^ bacteria/ml (McFarland 0.5). A 10^−6^ dilution of the suspension of McFarland 0.5 was then performed. An equal volume of drug-susceptible strains suspension in combination with MDR strains suspension from the same or different genotypes was prepared. Two Middlebrook 7H11 solid media were inoculated with 0.2 ml of bacterial suspension and two additionally selective Middlebrook 7H11 solid media (rifampicin + 1 mg/ml) were inoculated for strains in combination (cocultures). The cultures were incubated for 14 days at 37°C with 7% CO_2_. H37Rv (ATCC) laboratory referential strain (WT strain) was used as growth control. Blood agar was used to confirm strains’ suspension purity and was incubated for 72 h under the same conditions.

The number of strains generation and generation time was calculated according to the formulas^[5,18]^ indicated below.

- Number of generation: n = Log Nt− Log N0/Log 2
- Generation time: g = t/n → g = t Log 2/Log Nt− Log N0

g: strains generation time; n: Number of strains generation; Nt: Number of viable cells at final time t; N0: Number of viable cells at final initial time 0; t: total time of experience (14 days).

The relative competitive fitness (W) of strains was calculated using the following formula.^[5,19]^

- W = (ln [Rf ÷ Ri] ÷ ln [Sf ÷ Si])

W: Relative fitness of MDR train; Si and Ri: resistant and susceptible cells at T0 (baseline), respectively; Sf and Rf: resistant and susceptible cells at endpoint (day14), respectively.

- If W ˃ 1: mutant MDR strain more fit
- If W ˂ 1: mutant MDR strain less fit
- If W = 1: mutant MDR strain and WT have the same fitness.

### Statistical and data analysis

Data were processed and analyzed with Epi-info software (Epi Info™, V 7.2.2.6 CDC). The Student’s test was performed to compare the averages. Percentage comparisons were made by the Pearson’s *χ*^2^ test and Fisher’s exact test. *P* value was considered statistically significant when it was <0.05. The CRSLO scientific calculator (CS-82TL) was used for competitive fitness parameters calculations.

## Results

### Isolates included in the study

Two isolates from MTB T1, two MTB Beijing, and one MTB H37Rv were included in the study. We performed 24 competitive cultures (cocultures), and ten individual cultures and five blood agar cultures were included in final analysis.

### Number and time of generation of strains

In the individual strain culture, the number of generation of Beijing WT and MDR genotypes was 4.60 and 4.40, respectively, and that of H37Rv was 2.91. The number of generation of T WT and MDR genotypes were 2.69 and 2.37, respectively.

In coculture, the number of generation of H37Rv was 2.57 and 4.18, respectively, compared to its competitors MDR-genotypes T (1.72, rifampicin + HTm (Rif+HTm)) and Beijing (3.42, rifampicin + HBm (Rif+HBm)) [Table 1]. The generation number of WT T1 (TTm) was higher (2.16) compared to its drug resistant competitor (Rif + TTm) which was 1.75. The number of generation of WT Beijing (BBm) was 3.74 and that of its competitor was 3.67 [Table 1].

In individual culture, the mean generation time of the isogenic strains was 133.34 ± 8.44 h and 74.7 ± 1.66 h, respectively, for T, Tm and B, Beijing mutant (Bm); while they were 173.8 ± 18.2 h and 90.7 ± 0.86 h in isogenic cocultures, respectively, of T and Tm; B and Bm.

In individual strain cultures, T1 was grown slower (133.34 h average) compared to H37Rv (115.6 h) and Beijing (74.7 h average), and this difference was statistically significant (*P* < 0.01) [Table 1]. The mutant MR strains were grown slower than WT strains in both types of cultures, except TBm coculture (WT T1 and Beijing MR), where the Bm has a higher number of generation than that of his wild competitor (T1) [Table 1].

### Relative competitive fitness of strains

The mutant strains (m) were less competitive compared to that of WT strains (W < 1) in 5/6 of the cases or 83.3%. However, they were more competitive (W > 1) in 1/6 of cases (16.7%) [Table 2].

## Discussion

Our study evaluated the competitive fitness of WT and mutant strains on media with or without antibiotics in UCRC BSL-3 laboratory in Bamako. Strains of mycobacteria have been isolated from new or old TB patients. We found that the MDR strains were less competitive than their WT isogenics in general. Similar observations were made by Toungoussova *et al.* and Billington *et al*.^[19,20]^ However, one needs to point out that the mutant Beijing had a competitive fitness relatively close to that of its WT Isogen (W = 0.98) in our study, whereas Bhatter *et al.* reported a high competitive fitness of the mutant Beijing compared to its WT Isogen.^[5]^ This could be explained either by a low-cost MDR mutation or by acquisition of compensatory mutations, which was conducted specified in prior studies.^[4,11]^ In that case, it is better to sequence the strains of mycobacteria to identify the types of mutations, but this was not possible due to budget limitation.

In addition to mutation, the growth of strain could be influenced by the competitor. Thus, mutual inhibitions between competing strains by the production of biological substances have been reported.^[21]^ This would explain the reduced number of colony-forming units (CFU) of the competing strains (co-culture) compared to their CFU in individual culture in this study. However, another study^[22]^ found an increased number of CFU in cocultures.

### Some limitations of our study

Due to funding limitation, we were unable to carry out genotypic identification tests for rifampicin and isoniazid mutations, such as the Genotype® MTBDR plus test, or partial of whole-genome sequencing. In addition, we do not know if there will be a difference between fresh samples collected versus regrowth frozen samples in terms of fitness.

Despite these limitations, our study was the first of its kind to determine the fitness on different lineages of MTBc with both the resistant and pan-sensitive strains. The results are in line with previously published data and could be used in controlling the spread of DR-TB in Mali.

## Conclusion

This study found that both MTB T1 and MTB Beijing which developed MDRs were less competitive compared to their WT Isogen. That imply they are less competitive than drug susceptible strains in antibiotic free environment. That normally means, drug resistant strains spread slowly, so drug resistance mutation impacts bacteria competitive fitness in absence of treatment. This impact is called “Biological cost of mutation”. Thus, mutations could be an asset in the control of bacterial infections.

## Figures and Tables

**Table 1:** Number and time of generation of strains

Strains	iCFU	Final CFU	Strains generation number	Generation time (h) of strains	Mean±SD
Individual cultures of strains					
H	1.5×10^8^	11.27×10^8^	2.907	115.6	
T	1.5×10^8^	9.65×10^8^	2.69	124.9	133.34±8.44
Tm	1.5×10^8^	7.75×10^8^	2.37	141.78	
B	1.5×10^8^	36.325×10^8^	4.60	73.04	74.7±1.66
Bm	1.5×10^8^	31.75×10^8^	4.40	76.36	
Cocultures of strains					
HTm	1.5×10^8^	8.9×10^8^	2.57	130.73	163. 04±32.31
Tm	1.5×10^8^	4.95×10^8^	1.72	195.35	
HBm	1.5×10^8^	27.27×10^8^	4.18	80.38	89. 31±8. 93
Rif + HBm	1.5×10^8^	16×10^8^	3.42	98.24	
TTm	1.5×10^8^	6.68×10^8^	2.16	155.6	173.8±18.2
Rif + TTm	1.5×10^8^	5.05×10^8^	1.75	192	
TBm	1.5×10^8^	11.075×10^8^	2.88	116.7	99.525±17.17
Rif + TBm	1.5×10^8^	25.3×10^8^	4.08	82.35	
BTm	1.5×10^8^	18.3×10^8^	3.61	93.08	103.295±10.22
Rif + BTm	1.5×10^8^	11.7×10^8^	2.96	113.51	
BBm	1.5×10^8^	20.025×10^8^	3.74	89.84	90.695±0.86
Rif + BBm	1.5×10^8^	19.10×10^8^	3.67	91.55	

H: Drug susceptible H37Rv strain (in individual culture), T: Drug susceptible T1 strain in individual culture, B: Drug susceptible Beijing strain (in individual culture), Tm: Drug Beijing resistant T1 strain (in individual culture), Bm: Drug Beijing resistant Beijing strain (in individual culture), HTm: Drug susceptible H37Rv strain from the competition between H37Rv and Tm strains, HBm: Drug susceptible H37Rv from the competition between H37Rv and Bm strains, TTm: Drug susceptible T1 strain from the competition between T1 and Tm strains, TBm: Drug susceptible T1 strain from the competition between H37Rv and Bm strains, BTm: Drug susceptible Beijing strain from the competition between B and drug resistant Tm strains, BBm: Drug susceptible Beijing strain from the competition between B and Bm strains, Rif + HTm: Drug resistant T1 strain from the competition between H37Rv and Tm strains on selective media, Rif + HBm: Drug resistant Beijing strain from the competition between H37Rv and Bm strains on selective media, Rif + TTm: Drug resistant T1 strain from the competition between T and Tm strains on selective media, Rif + TBm: Drug resistant Beijing strain from the competition between T and Bm strains on selective media, Rif + BTm: Drug resistant T1 strain from the competition between B and Tm strains on selective media, Rif + BBm: Drug resistant Beijing strain from the competition between B and Bm strains on selective media

**Table 2:** Relative competitive fitness of mutants strains

Strains	iCFU	Final CFU	Relative fitness of strains (w)
HTm	1.5×10^8^	8.9×10^8^	0.67
Rif + HTm	1.5×10^8^	4.95×10^8^	
HBm	1.5×10^8^	27. 27×10^8^	0.81
Rif + HBm	1.5×10^8^	16×10^8^	
TTm	1.5×10^8^	6.68×10^8^	0.81
Rif + TTm	1.5×10^8^	5.05×10^8^	
BTm	1.5×10^8^	18.3×10^8^	0.82
Rif + BTm	1.5×10^8^	11.7×10^8^	
BBm	1.5×10^8^	20.025×10^8^	0.98
Rif + BBm	1.5×10^8^	19.10×10^8^	
TBm	1.5×10^8^	11.075×10^8^	
Rif + TBm	1.5×10^8^	25.3×10^8^	1.41

W: Relative fitness of strains, H: Drug susceptible H37Rv strain (in individual culture), T: Drug susceptible T1 strain in individual culture, B: Drug susceptible Beijing strain (in individual culture), Tm: Drug Beijing resistant T1 strain (in individual culture), Bm: Drug Beijing resistant Beijing strain (in individual culture), HTm: Drug susceptible H37Rv strain from the competition between H37Rv and Tm strains, HBm: Drug susceptible H37Rv from the competition between H37Rv and Bm strains, TTm: Drug susceptible T1 strain from the competition between T1 and Tm strains, TBm: Drug susceptible T1 strain from the competition between H37Rv and Bm strains, BTm: Drug susceptible Beijing strain from the competition between B and drug resistant Tm strains, BBm: Drug susceptible Beijing strain from the competition between B and Bm strains, Rif + HTm: Drug resistant T1 strain from the competition between H37Rv and Tm strains on selective media, Rif + HBm: Drug resistant Beijing strain from the competition between H37Rv and Bm strains on selective media, Rif + TTm: Drug resistant T1 strain from the competition between T and Tm strains on selective media, Rif + TBm: Drug resistant Beijing strain from the competition between T and Bm strains on selective media, Rif + BTm: Drug resistant T1 strain from the competition between B and Tm strains on selective media, Rif + BBm: Drug resistant Beijing strain from the competition between B and Bm strains on selective media
